# Association between ocular axial length and anthropometrics of Asian adults

**DOI:** 10.1186/s13104-021-05745-y

**Published:** 2021-08-26

**Authors:** Ari Shinojima, Toshihide Kurihara, Kiwako Mori, Yujiro Iwai, Akiko Hanyuda, Kazuno Negishi, Hidemasa Torii, Kazuo Tsubota

**Affiliations:** 1 Department of Ophthalmology, Keio University School of Medicine, 35 Shinanomachi, Shinjuku ku, Tokyo, 160-8582 Japan; 2 Laboratory of Photobiology, Keio University School of Medicine, 35 Shinanomachi, Shinjuku ku, Tokyo, 160-8582 Japan; 3 Keio University School of Medicine, 35 Shinanomachi, Shinjuku ku, Tokyo, 160-8582 Japan; 4 Tsubota Laboratory, Inc, 304 Toshin Shinanomachi-ekimae Bldg., 34 Shinanomachi, Tokyo, 160-0016 Japan

## Abstract

**Objective:**

The purpose of this study is to analyze axial length, body height, hand length, and foot length to find new factors that predict myopia and to identify gender differences as one of the factors of high myopia. A cross-sectional study was conducted as a single observation. Body height, hand length, and foot length were measured according to standard anthropometric methods. Axial length, retinal thickness, and choroidal thickness were measured using the IOL Master 700 and the Heidelberg Spectralis-OCT. To account for body height differences among participants, foot length/body height and hand length/body height were analyzed using a mixed-effects model.

**Results:**

A total of 80 eyes (men, n = 20, 40 eyes; women, n = 20, 40 eyes) were analyzed. The mean age was 33.5 years (range 21–59 years, SD: 9.6). For choroidal thickness, there was a significant association with axial length in men (p < 0.001) and a trend toward an association in women (p = 0.072). There was also a significant association between foot length/body height and axial length in men (p = 0.015), but not in women (p = 0.58). These results suggest that factors that determine body height and foot length may be related to axial length, although they vary by gender.

## Introduction

Myopia has been gradually increasing since around 1950 [1], and it is estimated that half of the world's population will be myopic by 2050 [2]. One of the characteristics of myopia is a long axial length (AL). It is generally known that longer AL results in thinner retina and choroid [3–5], and while some reports suggest that AL correlates with body height (BH) [6, 7], others suggest that AL does not correlate with BH [8].

In clinical practice, we sometimes encounter patients who are small in stature, such as children, but have a long AL. Therefore, we hypothesized that something other than BH is associated with AL. To the best of our knowledge, it is not well known whether AL is related to foot length (FL) or hand length (HL).

It has been reported that FL and HL correlate with BH [9]. In Japanese girls, the growth of FL is faster than that of BH until the age of 16, after which the growth of FL is slower than that of BH. Also, after the age of 13, the growth of HL is slower than the growth of BH. In Japanese boys, BH growth shows a similar growth pattern to that of FL and HL growth [10]. Thus, in boys and girls, the growth of FL and HL is known to be different from the growth of BH. Growth hormone (GH) is known to primarily promote longitudinal growth in children and adolescents, but has a variety of important metabolic functions throughout adulthood [11].

Since being female is a risk factor for high myopia [12, 13], we believe it would be valuable to know the gender differences. If new parameters related to AL and other body lengths are discovered, it may shed light on why there are gender differences in severe myopia.

## Main text

### Methods

A cross-sectional study was conducted between February 2020 and September 2020 at the Department of Ophthalmology, Keio University Hospital, Tokyo, Japan. All procedures were in accordance with the tenets of the Declaration of Helsinki. Written informed consent was obtained from all subjects prior to the study. This study was approved by the Ethics Committee of Keio University School of Medicine (Approval No. 20190241/UMIN000038975).

Subjects were healthy adults aged 20 years or older who offered to cooperate in this study. Exclusion criteria were as follows: those who could not give their consent to the study, those who had undergone eye surgery such as laser in-situ keratomileusis, those who were currently suffering from eye diseases such as uveitis, those who felt psychological distress when measuring physical parameters, and those who had a strong valgus toe.

Eighty eyes (men, n = 20, 40 eyes; women, n = 20, 40 eyes) were included in the study. Spectral-domain OCT (SPECTRALIS OCT; Heidelberg Engineering, Heidelberg, Germany) with EDI-OCT was used to measure foveal retinal thickness (RT) and subfoveal choroidal thickness (CT). AL was also measured using swept-source optical biometry (IOL Master 700; Carl Zeiss Meditec AG, Jena, Germany).

RT was defined as the distance from the inner limiting membrane to the inferior epithelial edge of the retinal pigment epithelium (RPE). CT was defined as the distance from the outer edge of the hyperreflective line corresponding to the RPE-Bruch's membrane complex to the inner edge of the choroid below the fovea. BH, FL and HL were measured according to standard anthropometric procedures [14, 15]. FL was defined as the linear distance from the most prominent part of the heel to the most distal part of the toe [16]. AL was measured automatically by detecting signals reflected from the cornea and RPE [17].

Descriptive data are presented as mean ± standard deviation (SD). FL and HL were analyzed as the relative value of FL to BH (FL/BH) and HL to BH (HL/BH), taking into account the difference in BH of the subjects according to the advice of the specialists of statistics. Statistical analysis was performed to determine the relationship between FL/BH, HL/BH, RT, CT and AL. Left and right eyes were considered as repeated measures, and statistics were performed using a mixed-effects model. Since the aim was to know the gender differences in factors affecting AL, we constructed a model that included the main effect of gender and the interaction of CT, RT, FL/BH, and HL/BH for each gender.

We chose "unstructured" for the repeated measures covariance in order to remove the intentional factor of whether or not a correlation was entered. The dependent variable was AL, factors were left and right, gender, and covariate data were CT, RT, FL/BH, and HL/BH. Fixed effects were gender only, gender x CT, gender x RT, gender x FL/BH, and gender x HL/BH, and their interactions were checked.

Statistical analysis was performed using SPSS (IBM® SPSS® Statistics, version 24). p-value < 0.05 was considered statistically significant.

### Results

Forty subjects, 80 eyes, met the inclusion criteria. The mean age was 33.5 years (range: 21–59 years, SD: 9.6). The participants were Japanese, Korean, and Chinese.

The differences in each parameter between men and women are shown in Table 1. AL, RT, HL, FL, and FL/BH were significantly different between men and women.

**Table 1:** Clinical profile of the 40 participants

	Men (n = 40 (R + L))	Women (n = 40 (R + L))	P value
AL (mm)	25.77 ± 1.39 (range 23.77–28.47)	25.16 ± 1.28 (range 23.03–28.17)	0.044*
RT (µm)	228.35 ± 15.44 (range 199–259)	215.30 ± 16.78 (range 159–252)	0.001*
CT (µm)	309.28 ± 137.28 (range 106–639)	276.20 ± 87.22 (range 123–491)	0.202
HL (cm)	18.31 ± 0.81 (range 17.0–19.7)	16.84 ± 0.88 (range 15.7–19.2)	< 0.001*
HL/BH	0.11 ± 0.01 (range 0.09–0.12	0.11 ± 0.01 (range 0.09–0.12)	0.583
FL (cm)	25.6 ± 1.09 (range 23.8–27.3)	22.9 ± 0.89 (range 21.6–24.8)	< 0.001*
FL/BH	0.15 ± 0.00 (range 0.14–0.15)	0.14 ± 0.00 (range 0.14–0.15)	< 0.001*

The symbol [asterisk] means a significant difference

**Fig. 1:**
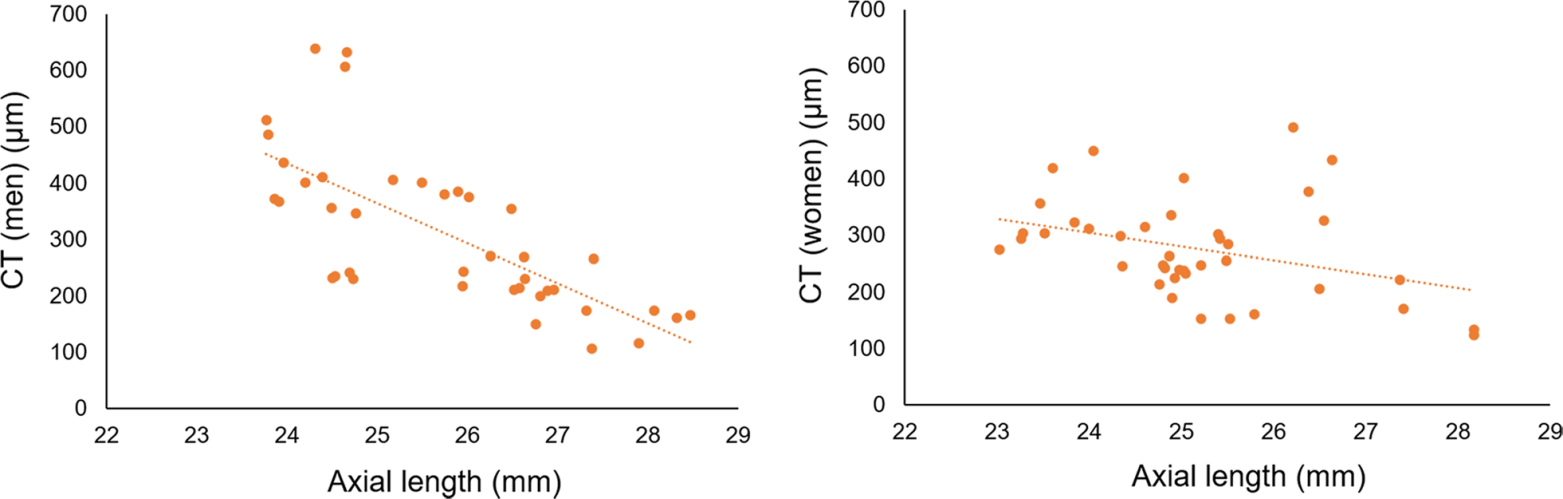
Association between AL and CT in men and women. For CT, there was a significant association with AL in men (p < 0.001) and a trend toward association with AL in women (p = 0.072)

**Fig. 2:**
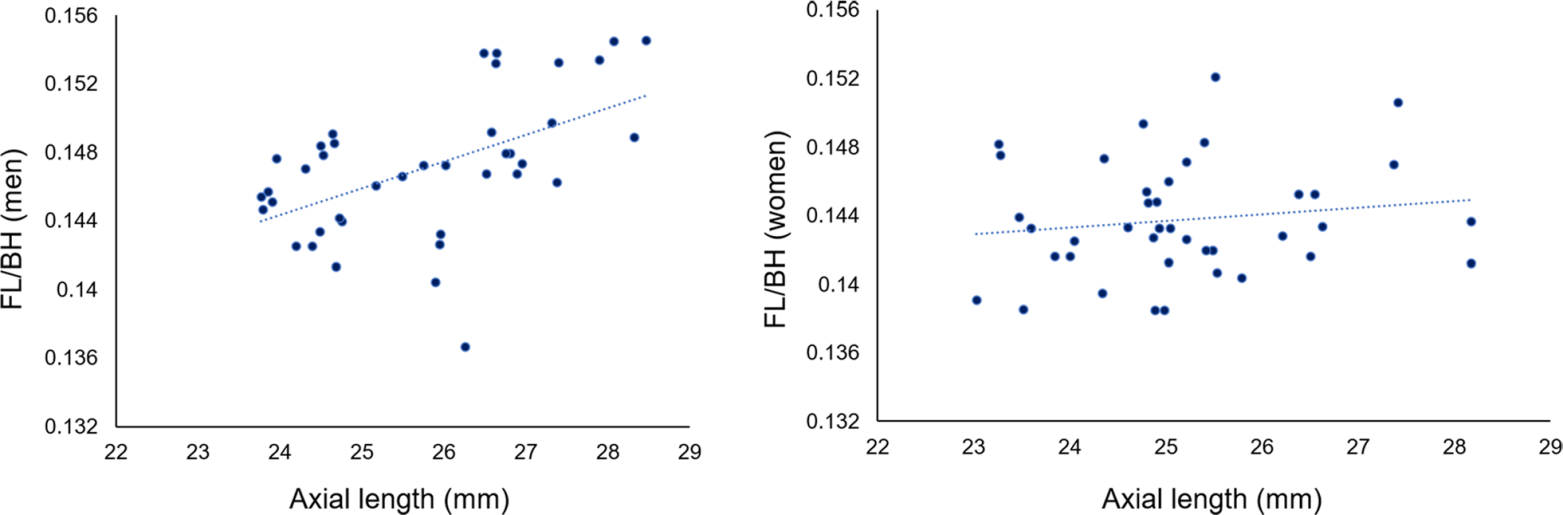
Association between AL and FL/BH in men and women. There was a significant association between FL/BH and AL in men (p = 0.015), but no significant association was found in women (p = 0.58)

The relationship between CT and AL by gender is shown in Fig. 1, and the relationship between FL/BH and AL by gender is shown in Fig. 2.

There was no significant association between AL and HL/BH in both men (p = 0.451) and women (p = 0.299) There was no significant association between AL and RT in both men (p = 0.555) and women (p = 0.447). There was no significant association between AL and age in both men (p = 0.617) and women (p = 0.611).

### Discussions

In this study, we chose hands and feet as the measurement parameters because they are simple to measure. When trying to measure the size of the liver as a parameter, it can vary because of adipose tissue depending on diet [18]. Moreover, when trying to measure the size of brain, it can also vary [19]. There are some reports BH correlates with spleen length [20] or kidney length [21]. However, measuring them requires echoes or other equipment, which is time-consuming.

In mixed gender reports, CT has been associated with AL [4, 5]. In the present study, CT was not significantly associated with AL in women. The number of participants was small and the variability may have been large. However, some have reported the gender difference [22, 23].

It has been reported that the peak refractive values of patients with GH deficiency tend to be hyperopic, while the peak mean refractive values of control healthy children tend to be myopic [24]. Therefore, we first focused on the possibility that taller participants may secrete more GH and have longer AL. In mixed-gender data, de Graaf et al. reported that GH therapy maintained equal growth in height and all body segments in children with severe growth retardation due to chronic renal failure with no signs of disproportionate growth [25]. On the other hand, Bannink et al. reported that girls with Turner syndrome were treated with GH therapy and grew proportionally except for FL [26].

Rickert, et al. suggested that direct injection of recombinant GH and application of low-intensity pulsed ultrasound around the distal femoral physis in rabbits may have a positive effect on microscopic growth without short-term adverse sequelae [27]. Recently, AL in a group of acromegaly patients did not show any significant difference from the control group [28]. Therefore, it is necessary to consider not only GH, but also other factors. Unknown factors, such as low-intensity pulsed ultrasound, may affect bone growth in humans. Similarly, unknown factors may be influencing human ocular axial lengthening. In humans, the amount of TGF-β2 in the aqueous humor is significantly correlated with AL [29] and TGF-β is increased by GH [30]. Female growth factors belonging to the TGFβ superfamily are also expressed developmentally in ovarian somatic cells and oocytes, and have been found to function as intrafollicular regulators of folliculogenesis [31]. Possibly the difference in TGF-β levels between men and women may have some effect on AL. Further study is needed to ascertain the additional factors related to AL.

One of the strengths of our study is that we found that the association between FL/BH and AL is appropriate for men. This is the first report of its kind, to our knowledge. It is reported that AL (eyeball) tends to grow up to 16–18 years of age [32]. Growth velocity approaches zero at age 18 in boys and at age 16 in girls [33]. In this study, healthy adults over the age of 21 were analyzed. The sample is considered to have been collected appropriately to investigate the correlation between AL and hands or feet.

In our study, Chinese, Korean and Japanese participated. Eighty years ago, 10–20% of the Hong Kong or Taiwan population was short-sighted [1]. We believe not only ethnicities, but also other factors such as environmental factors, foods, nearby work, etc., may have been influenced for myopia.

In conclusion, there was a significant association between FL/BH and AL in men, suggesting that factors determining each body size may be different in men and women, although the possibility remains that factors determining BH, FL, and HL are related to AL.

### Limitations

- Only adults aged 20 years and older, only Asian data were included.
- Only the association between AL and each body part at some points were analyzed.
- No aqueous humor tests, blood tests, or genetic tests were performed.
- No long-term follow-up and small number of participants were included.

## Data Availability

Not applicable.

## Abbreviations

AL: Axial length
BH: Body height
FL: Foot length
HL: Hand length
RT: Retinal thickness
CT: Choroidal thickness
